# 

**Published:** 1887-07

**Authors:** 


					﻿idvcts. a copy.
JULY, 1887.
$i.oo per year.
I.L-MI'S
ejoVRNAL'J
HEALTH
ESTABLISHED 18^4
U—• 34
#£• 7..
OFFIOE: 206 BROADWAY. ETCHING POST BUILDING, Bonm 11.JL T.
Entered as Second-class Matter at the Post-Office, New York.
				

